# Role of Epiregulin on Lipopolysaccharide-Induced Hepatocarcinogenesis as a Mediator via EGFR Signaling in the Cancer Microenvironment

**DOI:** 10.3390/ijms25084405

**Published:** 2024-04-17

**Authors:** Takahiro Kubo, Norihisa Nishimura, Kosuke Kaji, Fumimasa Tomooka, Akihiko Shibamoto, Satoshi Iwai, Junya Suzuki, Hideto Kawaratani, Tadashi Namisaki, Takemi Akahane, Hitoshi Yoshiji

**Affiliations:** Department of Gastroenterology, Nara Medical University, 840, Shijo-cho, Kashihara 634-8522, Japan

**Keywords:** epiregulin, tumor microenvironment, lipopolysaccharides, interleukin-8, tumor angiogenesis

## Abstract

Lipopolysaccharides (LPSs) have been reported to be important factors in promoting the progression of hepatocellular carcinoma (HCC), but the corresponding molecular mechanisms remain to be elucidated. We hypothesize that epiregulin (EREG), an epidermal growth factor (EGF) family member derived from hepatic stellate cells (HSCs) and activated by LPS stimulation, is a crucial mediator of HCC progression with epidermal growth factor receptor (EGFR) expression in the tumor microenvironment. We used a mouse xenograft model of Huh7 cells mixed with half the number of LX-2 cells, with/without intraperitoneal LPS injection, to elucidate the role of EREG in LPS-induced HCC. In the mouse model, LPS administration significantly enlarged the size of xenografted tumors and elevated the expression of EREG in tumor tissues compared with those in negative controls. Moreover, CD34 immunostaining and the gene expressions of angiogenic markers by a reverse transcription polymerase chain reaction revealed higher vascularization, with increased interleukin-8 (IL-8) expression in the tumors of the mice group treated with LPS compared to those without LPS. Our data collectively suggested that EREG plays an important role in the cancer microenvironment under the influence of LPS to increase not only the tumor cell growth and migration/invasion of EGFR-positive HCC cells but also tumor neovascularization via IL-8 signaling.

## 1. Introduction

Hepatocellular carcinoma (HCC) is the most common primary malignancy of the liver and a major global health concern [1]. It is characterized by its aggressive nature, rapid progression, and high mortality rates. HCC typically arises in the setting of chronic liver diseases, including viral hepatitis, alcohol-related liver disease, non-alcoholic fatty liver disease, and cirrhosis [2,3,4]. The incidence of HCC has been steadily rising worldwide, posing significant challenges for early detection, accurate diagnosis, and effective treatment strategies. Understanding the underlying molecular mechanisms and key signaling pathways involved in HCC development and progression is crucial for the development of novel targeted therapies and improved clinical outcomes.

Lipopolysaccharides (LPSs) derived from the intestine have been reported as a major factor in the progression of liver fibrosis through Toll-like receptor 4 (TLR4) signaling [5]. LPS is a potent inducer of the activation of hepatic stellate cells (HSCs) via binding to TLR4, and this can strongly drive liver fibrosis progression and, consequently, liver cirrhosis, fostering a background for HCC progression [6]. It has also been reported that LPS administration can induce hepatocarcinogenesis in an experimental mouse model [7]. However, the concise mechanisms of LPS on HCC progression remain to be elucidated.

A previous study revealed that activated HSCs secrete epiregulin (EREG) when stimulated with LPS [8]. LPS binding to TLR4 induces the activation of nuclear factor-kappa B (NF-κB) signaling leading to EREG production. This mechanism has also been indicated in colonic cells. EREG is a multifunctional cytokine belonging to the epidermal growth factor (EGF) family that plays crucial roles in various physiological and pathological processes [9]. Initially identified as a potent mitogen for epithelial cells, EREG has since been recognized for its pleiotropic effects on cell proliferation, survival, migration, and differentiation [10,11,12]. Moreover, emerging evidence suggests that EREG is implicated in the regulation of tissue homeostasis, wound healing, and immune responses [13,14]. In recent years, the dysregulation of EREG expression has been implicated in several diseases, including cancer, inflammation, and tissue remodeling [15,16]. The role of EREG on cancer development has been demonstrated in various cancers such as breast cancer, lung cancer, and colorectal cancer. Elevated EREG expression activates epidermal growth factor receptor (EGFR) signaling pathways that promote cell proliferation in bladder cancer [17]. EREG enhanced the invasive and proliferative activities of non-small cell lung cancer cell lines [18]. A high EREG expression promotes migration and invasion through the activation of protein kinase B (AKT) and extracellular signal-regulated kinase (ERK) signaling in salivary adenoid cystic carcinoma cells [19]. In cases of HCC, it has been shown that EREG knockdown can inhibit disease progression [8].

Recently, increasing attention has been directed toward the tumor microenvironment (TME) to derive novel therapeutic targets against various cancers. TME comprises various cell types, including immune cells, fibroblasts, and endothelial cells, as well as extracellular matrix components and signaling molecules [20]. Cellular interaction between the aforementioned cells have also been reported to induce HCC progression due to immunosuppression, migration/invasion, cancer heterogeneity, and angiogenesis [21]. Interestingly, it has been reported that increased EREG expression by Mucin-1 (MUC1) deficiency in fibroblasts and epithelial cells accelerated lung cancer development through the EGFR/AKT pathway [22]. EREG has also been suggested that it may be involved in the paracrine regulation of colitis-associated neoplasms, because EREG stimulates the proliferation of adjacent intestinal epithelial cells leading to their development into tumor cells [23].

Based on the above evidence, we hypothesize that HSC-derived EREG is a mediator for HCC progression caused by LPS stimulation in the TME. In this study, we demonstrated the possible mechanisms of EREG pertaining to LPS-induced hepatocarcinogenesis, which may lead to novel therapeutic strategies against HCC.

## 2. Results

### 2.1. LPS Induces the Production of EREG from LX-2 Cells In Vitro

**Figure 1:**
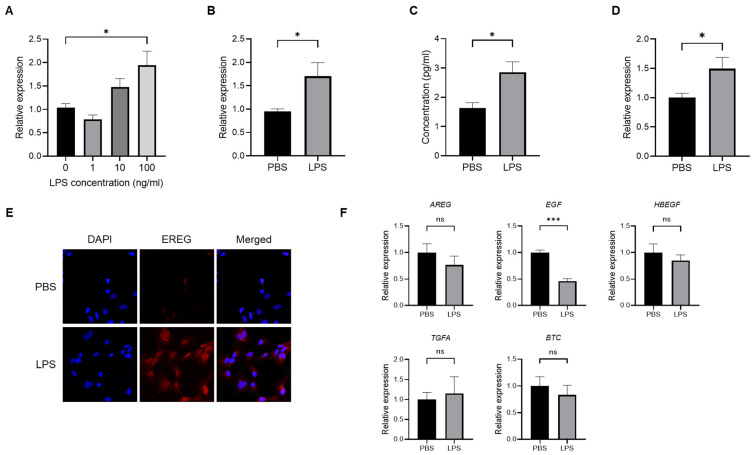
Change in *EREG* expression and production in LX-2 cells by LPS stimulation. (**A**) Expression level of *EREG* in LX-2 stimulated with different concentrations of LPS (0, 1, 10, and 100 ng/mL) measured by RT-PCR. (**B**) Expression of *EREG* in LX-2 cells measured by RT-PCR and (**C**) production of EREG protein level by enzyme-linked immuno-sorbent assay (ELISA) in the supernatant with LX-2 cells cultured with/without 100 ng/mL of LPS for 24 h. (**D**) *EREG* expression in LX-2 cells stimulated with/without recombinant human EREG protein (100 ng/mL) by RT-PCR. (**E**) Representative images of immunostaining for EREG in LX-2 cells compared between the conditions stimulated with/without LPS stimulation. Magnification, 60×. (**F**) Gene expression levels of other members in the EGF family in LX-2 cells with/without LPS stimulation. Graphs showed the mean ± SEM. *; *p* < 0.05, ***; *p* < 0.001, ns; not significant.

To confirm the production of EREG from HSCs, we first stimulated LX-2, which is an HSC line, with different concentrations of LPS (0, 1, 10, and 100 ng/mL) to investigate the change in *EREG* expression. As shown in Figure 1A, the *EREG* expression in LX-2 has increased in the group with the higher LPS concentration. Similarly, when LX-2 cells were stimulated with 100 ng/mL of LPS, both the expression and production of EREG increased compared with those in negative controls (Figure 1B,C). The increased *EREG* expression in HSCs by LPS stimulation was also revealed in immunostaining (Figure 1E). Moreover, EREG itself can induce *EREG* expression, as shown in Figure 1D. We measured the change in expression of other factors in the EGF family, including amphiregulin (*AREG*), EGF, heparin-binding EGF-like growth factor (*HBEGF*), transforming growth factor alpha (*TGFA*), and betacellulin (*BTC*), in LX-2 cells. The expressions of these genes except *EGF* showed no significant differences between the LPS administration and negative control groups (Figure 1F). The *EGF* expression showed significant differences between both groups, but it was decreased in the LPS-treated group compared to the PBS group.

### 2.2. Administration with LPS Accelerates the Development of Huh7/LX-2 Xenografted Tumors In Vivo

**Figure 2:**
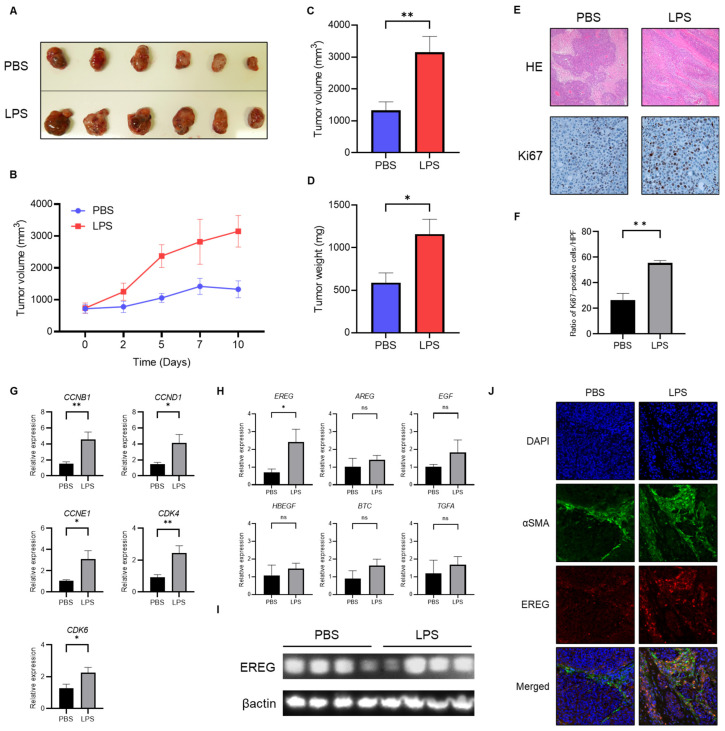
Administration of LPS enhances the development of Huh7 tumors mixed with LX-2 cells in vivo. (**A**) The image of xenografted tumors harvested from *BALB-C nu/nu* mice with/without LPS administration. Above: PBS group (negative control); below: LPS group. (**B**) Change in the average tumor volume in the PBS- and LPS-treated groups through the time course during the observation period. Average of (**C**) tumor volume and (**D**) tumor weight in both the PBS and LPS groups at the end of the experiment. (**E**) Representative images of hematoxylin and eosin (H&E) staining and Ki-67 immunostaining in tumor tissue harvested from mice in the PBS- and LPS-treated groups. Magnifications: 20× for H&E and 40× for Ki-67 staining. (**F**) Semiquantitative analysis for the cell number of Ki67-positive cells in the tumor tissue of both groups. (**G**) Expressions of cell-cycle markers in a Huh7+LX-2 mixed tumor measured by RT-PCR. (**H**) The gene expression levels of the EGF family members comprising EREG in both groups were measured by RT-PCR. (**I**) Western blotting analysis for *EREG* expression in tumor tissues in both groups with/without LPS administration. (**J**) Representative images of αSMA and EREG immunostaining in tumor tissue harvested from mice in the PBS- and LPS-treated groups. Magnifications: 20× for αSMA and EREG staining. Data are presented as mean ± SEM. *; *p* < 0.05, **; *p* < 0.01, ns; not significant.

We subsequently investigated the role of EREG in liver cancer by administering LPS in vivo. To develop this tumorigenesis model, we cultured Huh7 cells, followed by LX-2 cells, and, subsequently, mixed and xenografted them in the back of *BALB-C nu/nu* mice subcutaneously. Thereafter, an intraperitoneal injection with/without LPS (0.5 mg/kg) twice a week for 3 weeks after confirming engraftment was performed. It was revealed that the volume of xenograft tumors of Huh7/LX-2 cells in the LPS administration group was significantly higher than that in the control group (Figure 2A–C). Similarly, the average weight of tumors in the LPS-treated group was markedly higher than that in the negative group (Figure 2D). Immunostaining analysis demonstrated that the number of Ki67-positive tumor cells was significantly higher in the LPS group than in the phosphate-buffered saline (PBS) group (Figure 2E,F). The expression level of cell-cycle markers, including cyclin B1 (*CCNB1*), cyclin D1 (*CCND1*), cyclin E1 (*CCNE1*), cyclin-dependent kinase 4 (*CDK4*), and cyclin-dependent kinase 6 (*CDK6*), as measured by RT-PCR, was elevated in the LPS group compared to the untreated control (Figure 2G). As previously described, CCNB1 is a key initiator of mitosis that leads to cell-cycle progression, and CCND1 plays a crucial role in driving the G1-S transition of the cell cycle. Likewise, CCNE1 also stimulates cell proliferation. Therefore, the upregulation of these cyclins indicated the promotion of the cell proliferation of tumor cells. In addition, CDK4/6 is required for the G1-S transition. The upregulation of CDK4/6 is observed, meaning that LPS promotes cell proliferation in tumors. Next, we compared the *EREG* expression in tumors in both groups. As shown in Figure 2H, among members of the EGF family, only the *EREG* expression in tumor tissue showed a higher expression in the LPS-treated group than in the negative control. In addition, a Western blotting (WB) analysis demonstrated a higher expression of EREG in the LPS-treated group than in the negative control (Figure 2I). We confirmed that activated HSCs stimulated with LPS expressed a high *EREG* expression in tumor tissue (Figure 2J). LX-2 detected by alpha-smooth muscle actin (αSMA) staining showed a higher expression level of EREG in tumor tissue in the LPS-treated group than that in the PBS group, suggesting that activated HSCs are the major source of EREG in these xenografted tumors. 

### 2.3. EREG Promoted Cell Proliferation, and Migration/Invasion Activity of Liver Cancer Cells Expressing EGFR In Vitro

**Figure 3:**
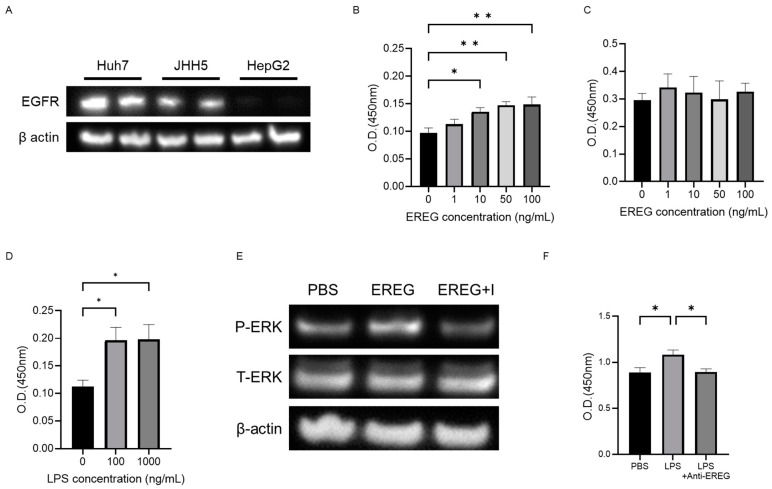
EREG promoted cell proliferation and migration/invasion activity of liver cancer cells expressing EGFR in vitro. (**A**) Expression levels of *EGFR* in each HCC line (Huh7, JHH, and HepG2). *EGFR* was expressed in Huh7 and JHH cells but not in HepG2 cells. Cell proliferation of (**B**) Huh7 cells and (**C**) HepG2 cells was stimulated with different concentrations of EREG (0, 1, 10, 50, and 100 ng/mL). (**D**) Cell proliferation of Huh7 co-cultured with LX-2 was measured by the WST-1 assay when stimulated with 100 and 1000 ng/mL of LPS. (**E**) Change in the phosphorylation of ERK1/2 in Huh7 stimulated with EREG and in combination with EREG and EGFR inhibitors (AG1478; 100 ng/mL). (**F**) Cell proliferation of Huh7 co-cultured with LX-2 with LPS stimulation and a combination of LPS and anti-EREG antibodies. Significance was determined using a one-way analysis of variance for group comparisons. *; *p* < 0.05, **; *p* < 0.01.

In what follows, we study the effects of EREG on the progression of HCC using Huh7, JHH-5, and HepG2. As shown in Figure 3A, Huh7 and JHH5 have EGFR, whereas HepG2 has a relatively low level of *EGFR* expression, as described in previous papers [24].

Using the water-soluble tetrazolium-1 (WST-1) assay, it was revealed that the cell proliferation of Huh7 was increased with the concentration of recombinant EREG (rEREG) in a dose-dependent manner (Figure 3B). JHH5 showed a similar pattern as seen in Huh7. In contrast, external stimulation with rEREG did not affect the cell proliferation of HepG2 (Figure 3C). Conditioned media derived from LX-2 with LPS supplementation showed a significant increase in the cell proliferation of Huh7 compared with that in the untreated control (Figure 3D). A WB analysis also revealed that stimulation with rEREG induced the phosphorylation of ERK in Huh7, which is a crucial signaling pathway for cell proliferation that was inhibited by an EGFR inhibitor (AG1478; 100 ng/mL) (Figure 3E). Furthermore, we investigated the role of EREG on LPS-induced tumor cell proliferation; we measured the cell proliferation rate of Huh7 cells co-cultured with LX-2 cells treated with/without anti-EREG antibodies under LPS stimulation. As shown in Figure 3F, anti-EREG antibodies significantly reduced the cell proliferation of Huh7 by LPS stimulation, suggesting that EREG plays a crucial role in LPS-induced tumor development.

### 2.4. EREG Drives the Cell Migration and Invasion Activity of Liver Cancer Cells with EGFR

**Figure 4:**
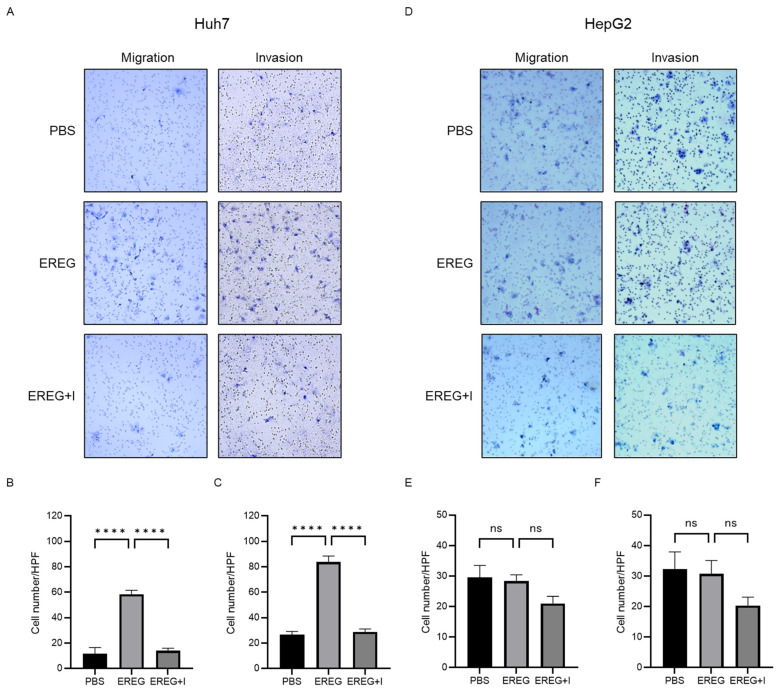
EREG drives the migration and invasion activities of only liver cancer cell lines with EGFR. (**A**) Representative images of migration and invasion assays in Huh7 cells when stimulated with EREG only and both EREG and EGFR inhibitors. The average cell number of Huh7 cells (**B**) migrated and (**C**) invaded in each group. (**D**) Representative images of migration and invasion assay in HepG2 cells under EREG stimulation or with both EREG and EGFR inhibitors. The average number of (**E**) migrated and (**F**) invaded HepG2 cells in each group. Magnification, 20×. Significance was determined using a one-way analysis of variance for group comparisons. **** *p* < 0.0001, ns; not significant.

Next, we elucidated the effect of EREG on the cell migration/invasion activity of HCC lines. Huh7 cells, which have abundant EGFRs, showed a significant increase in migrated and invaded cells by the administration of rEREG (Figure 4A–C). EGFR inhibitor AG1498 significantly inhibited the migration and invasion of Huh7 cells induced by EREG stimulation. On the other hand, HepG2 cells, which have no expression of EGFRs, did not migrate and invade the lower compartment (Figure 4D–F). 

### 2.5. EREG Promotes the Production of Interleukin 8 (IL-8) from Cancer Cells through the Expression of EGFR

**Figure 5:**
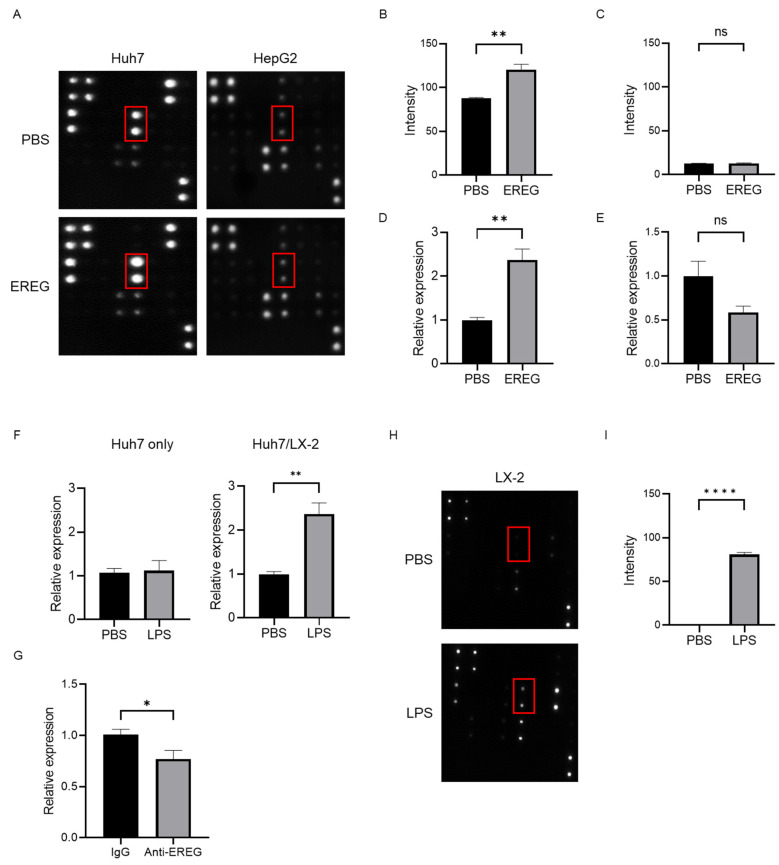
EREG increased IL-8 production from cancer cells through EGFR signaling to induce tumor neovascularization. (**A**) Representative images of the change in angiogenic factors in both Huh7 and HepG2 cells with/without stimulation with EREG. IL-8 levels in Huh7 cells only increased during stimulation with rEREG. Red box showed the expression of IL-8. The graph for the intensity of IL-8 in the angiogenesis array kit in the untreated and EREG-treated groups (**B**) in Huh7 and (**C**) in HepG2. The graph for the expression level of *IL-8* by RT-PCR in the untreated and EREG-treated groups (**D**) in Huh7 and (**E**) in HepG2. (**F**) The expression level of *IL-8* in Huh7 cells in the monoculture group (left graph) and the group co-cultured with LX-2 (right graph) under the LPS stimulation. (**G**) Change in *IL-8* expression levels when treated with/without anti-EREG antibodies under LPS present. (**H**) Representative images of the change in angiogenic factors in LX-2 with/without stimulation with LPS. (**I**) The graph for the intensity of IL-8 measured by the angiogenesis array kit in the untreated and LPS-treated groups in LX-2. Significance was determined using a one-way analysis of variance for group comparisons. * *p* < 0.05, ** *p* < 0.01, **** *p* < 0.0001, ns; not significant.

To investigate the mechanisms underlying the cancer progression induced by EREG, we focused on angiogenesis. We used an array kit that can detect various angiogenic factors to find which factors were changed with/without the stimulation with EREG. Among these, EREG induced the expression of IL-8 only in Huh7 that had the expression of *EGFR* (Figure 5A,B). The analysis using this angiogenesis array kit demonstrated that Huh7 did not change the level of other growth factors including vascular endothelial growth factor (VEGF), EGF, and platelet-derived growth factor (PDGF-BB). Similarly, a reverse transcription polymerase chain reaction (RT-PCR) showed that the IL-8 expression in Huh7 was higher in EREG-treated cells than in negative controls (Figure 5D). In contrast, in HepG2 cells, EREG did not change the expression of angiogenic factors, including IL-8, measured by both array kits and RT-PCR (Figure 5A,C,E). Thereafter, we co-cultured Huh7 cells with LX-2 using Transwell plates to investigate the cellular interaction between HCC cells and HSCs with LPS present. As shown in Figure 5F, LPS did not affect the IL-8 expression when Huh7 cells were mono-cultured, whereas the expression of IL-8 in Huh7 increased when it was co-cultured with LX-2 cells, suggesting that cellular interaction between Huh7 cells and LX-2 cells was important to producing IL-8. Furthermore, IL-8 induction was inhibited when the co-culture of Huh7 cells with LX-2 cells was treated with anti-EREG antibodies with LPS present (Figure 5G). In addition, we checked the changes of these angiogenic factors produced by LX-2 under LPS stimulation. As shown in Figure 5H, we found that LX-2 increased the production of IL-8, monocyte chemotactic Protein-1 (MCP-1), growth-related oncogene (GRO), and tissue inhibitor of metalloproteinases 2 (TIMP-2), but not those of VEGF family members. The elevation of MCP-1 and TIMP-2 is consistent with previous reports because they play important roles in liver fibrogenesis when HSCs are activated by LPS stimulation [25]. The change in the intensity of IL-8 production from LX-2 showed the significant difference between the untreated group and the LPS-treated group (Figure 5I).

### 2.6. Elevation of IL-8 Is Closely Linked to the Progression of Neovascularization in Tumor Tissue

**Figure 6:**
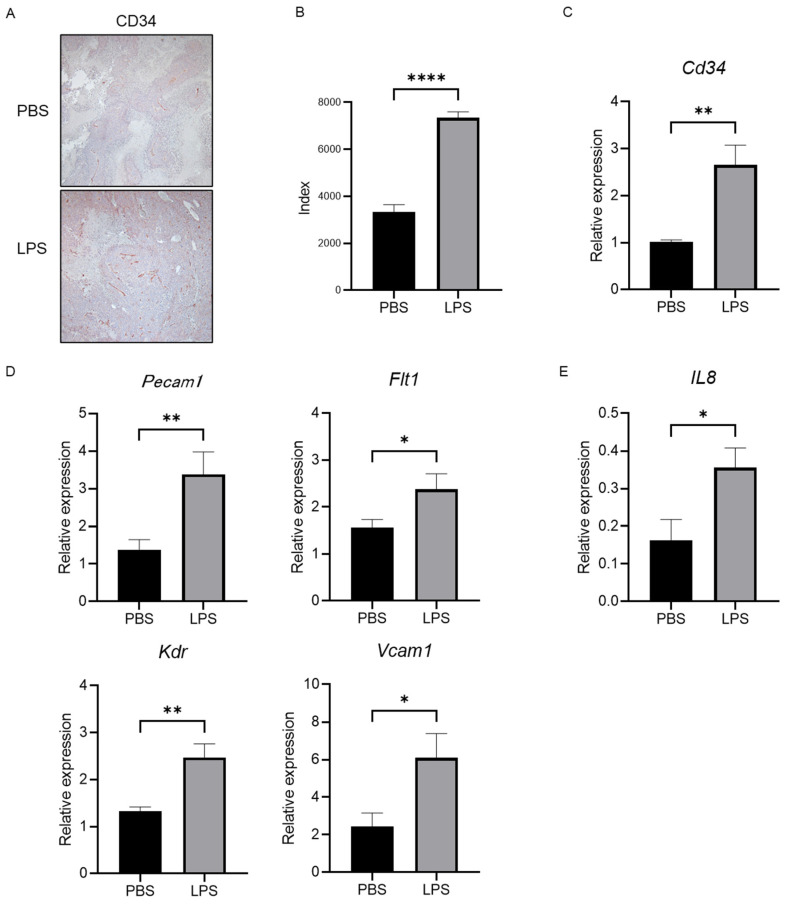
Tumor neovascularization and levels of angiogenic factors are promoted in the LPS-treated group, along with increased *IL-8* levels in the tumor tissue. (**A**) Representative images of CD34 immunostaining in xenografted tumor tissue in both the negative control and LPS-injected groups. (**B**) Semiquantitative analysis for the measurement of the CD34-positive area in the tumors. The LPS-treated group showed a significant increase compared with the negative control group. (**C**) Gene expression of *Cd34* was measured by RT-PCR in tumor tissue in both groups. (**D**) The expression levels of angiogenic markers were measured by RT-PCR. (**E**) Gene expression of *IL-8* was investigated by RT-PCR. Significance was determined using a one-way analysis of variance for group comparisons. * *p* < 0.05, ** *p* < 0.01, **** *p* < 0.0001.

To elucidate the change in tumor vascularization, we performed CD34 immunostaining in xenografted tumor tissue. As shown in Figure 6A,B, we found that LPS increased the positive area of CD34 in the tumors compared with the group with PBS injection as the negative control. The expression level of *Cd34* measured by RT-PCR also showed the same pattern as seen by immunostaining (Figure 6C). We additionally investigated the expression levels of angiogenic markers, including *Pecam1*, *Flt1*, *Kdr*, and *Vcam1*. All cells suggested a significant increase in neovascularization in the tumor in a group with LPS administration compared with the negative controls (Figure 6D). Furthermore, the expression level of *IL-8* was higher in the tumors in the mice group injected with LPS than in that in mice with a PBS injection (Figure 6E), suggesting that LPS accelerated the development of tumor neovascularization via IL-8 elevation.

## 3. Discussion

It is well-known that LPS plays a crucial role in the development of liver fibrosis and, consequently, liver cirrhosis. Moreover, it has been reported that LPS can accelerate HCC progression [7]. However, the molecular mechanisms of LPS related to hepatocarcinogenesis have not been characterized. As recent studies have focused on cellular interaction in the TME [26], we focused on EREG, an EGF family member that may be a mediator for LPS-induced liver cancer development, in this study. Although EREG knockout inhibits HCC tumor growth, the molecular mechanisms of EREG remain unclear [8]. Our data demonstrated that EREG acts as a mediator for cellular interactions between HCC and activated HSCs to accelerate HCC development by inducing cancer cell proliferation, migration/invasion, and tumor neovascularization during stimulation with LPS.

In this study, we first confirmed using an in vivo study that LPS administration promotes the growth of HCC/HSC-mixed tumors, as previously described [27]. In the tumor tissue stimulated with LPS, the EREG expression significantly increased compared with that in negative controls, as seen in previous studies [8]. In vitro studies corroborated that EREG can directly promote the proliferation of Huh7 cells, which have an abundant expression of *EGFR* [24]. EGFR signaling is a key mechanism for stimulating cell proliferation. EGFR activation initiates intracellular pathways that, in turn, promote cellular DNA replication and division [28]. The EGFR signaling pathway is often aberrantly activated in cancer cells and contributes to unregulated cancer cell proliferation and tumor growth [29]. Since stimulation with rEREG did not increase the proliferation of HepG2 cells, which have low expression levels of EGFR, the effect of EREG is thought to be specifically dependent on EGFR signaling. *EREG* has been indicated to be expressed in several HCCs, and the increase in *EREG* expression in liver cancer cells induces cell proliferation activity. The knockdown of *EREG* and *N-ras* in an HCC line inhibited their cell proliferation, migration, and invasion [30]. In our study, the external stimulation with rEREG also promoted the cell proliferation of liver cancer cells, suggesting that the paracrine effect of EREG additionally drives the malignant potential of cancer cells.

EREG also showed an increase in the cell migration and invasion of cancer cell lines with the abundant expression of EGFR in our study. A previous study revealed that activated HSCs promote epithelial mesenchymal transition (EMT) upregulation in hepatic cancer cells [31]. The activation of EGFR signaling is closely associated with cellular interaction between cancer cells and the surrounding cells, which can induce the migration and invasion of cancer cells, including lung, colon, and gastric cancer cells and HCC [32,33,34,35]. Similarly, EREG-overexpressed esophageal cancer cells demonstrated promoted migration and invasion [36]. In contrast, the secretion of EREG from intestinal epithelial cells is less than that from HSCs [37]. Therefore, the crosstalk between HCCs and HSCs is more important for the progression of HCC.

We further studied tumor angiogenesis because it is also associated with HCC progression. EREG has been reported to promote the proliferation of vascular smooth muscle cells and vascular remodeling [38]. Although many angiogenic factors have been previously described [39,40], the major angiogenic factor for EREG-induced neovascularization remains to be elucidated. Therefore, we explored which angiogenic factors were elevated by the stimulation with EREG. Our study using angiogenesis array kits revealed that the production of IL-8 was promoted by EREG in Huh7 cells, which have high levels of *EGFR* expression. In contrast, HepG2 cells without *EGFR* expression did not show any changes in angiogenic factors. This angiogenesis array kit can analyze other important angiogenic factors including EGF, VEGF, and PDGF-BB. Our investigation indicated EREG specifically induced the IL-8 production, but not other factors in Huh7, meaning that IL-8 signaling is the main pathway for EREG-induced angiogenesis. As LX-2 is also observed to secrete IL-8 with LPS present, HSCs have a direct and an indirect effect on the promotion of tumor angiogenesis via IL-8 signaling.

IL-8 has been reported as a potent inducer for tumor angiogenesis [41]. EGF stimulates EGFR to increase the production of IL-8 from cancer cells [42]. A recent study has demonstrated that LPS also promotes angiogenesis via the TLR4 pathway [6], meaning that EREG is a candidate for the mediator of LPS-induced angiogenesis. IL-8 can be secreted by fibroblasts, cancer-associated fibroblasts (CAFs), endothelial cells, epithelial cells, dendritic cells, monocytes, macrophages, and cancer cells [43]. IL-8 secretion from CAFs induces monocyte infiltration that leads to tumor-associated monocytes and suppresses the natural killer cell’s function in colorectal cancer [44]. Angiogenesis induced by IL-8 contributes to tumor progression in various cancers, including salivary adenoid cystic carcinoma and pancreatic cancer [45,46]. IL-8 has also been reported to possibly promote cell migration and HCC invasion via the induction of EMT [47]. Furthermore, our in vivo study showed higher vascularization in tumor tissue along with an increased IL-8 expression, as shown in previous studies.

Moreover, EREG can be a potential marker to predict the prognosis of patients with HCC. It has been reported that the elevation of serum IL-8 levels may predict a poor prognosis in patients with HCC [48]. Similarly, as previous papers have reported that the higher expression of EREG could predict a poor prognosis among patients with HCC, IL-8 signaling may be considered a major pathway for the development of EREG-induced HCC.

This study had several limitations. First, we have not investigated the influence of other EGF family members, although LX-2 mainly increases the production of EREG under LPS stimulation. As other factors in the EGF family can also bind to EGFR, we may need to compare the efficacy of EREG and other members, including EGF. Likewise, we have not investigated about the influence of other growth factors except the EGF family. As HSCs can release other growth factors like VEGF and PDGF-BB, which are also inducers for tumor growth and tumor angiogenesis, we need further investigations to check their changes and efficacies. Second, the role of EREG in the interaction between cancer cells and other non-parenchymal cells, such as liver sinusoid endothelial cells and Kupffer cells, remains unknown, as we focused on the relationship between HCCs and HSCs because EREG was mainly produced by HSCs [8]. Third, we investigated the effect of EREG on tumor development by using only several HCC lines with different levels of EGFR expression. As EREG selectively binds to EGFR, it may not effective against HCC with few EFGR expression. However, a previous study revealed that the overexpression of EGFR was observed in approximately 70% of HCC patients, and EGFR overexpression is associated with the occurrence of metastasis, poor survival rate, and the aggressiveness of tumors. Moreover, EGFR activation could contribute to drug resistance against the TKI inhibitor, Lenvatinib [24], suggesting that inhibition of the EREG may improve the efficacy of Lenvatinib. Fourth, our in vivo study demonstrated the cascade from LPS stimulation on HSCs to tumor development through the elevation of EREG and IL-8 expression in the xenografted tumor tissue, which is the main purpose of our study design, but it is not perfect to support the identification of the specific effects of EREG on cancer development. It may require further animal experiments, such as specific pharmacological inhibition or the genetic knockdown of *EREG*, to expand the role of EREG in LPS-associated HCC progression.

## 4. Materials and Methods

### 4.1. Experimental Mouse Model

Six-week-old male BALB/c nu/nu mice were purchased from Japan SLC (Shizuoka, Japan). LX-2 and Huh7 were cultured separately and, subsequently, mixed in a 2:1 fashion (Huh7: 1 × 10^7^ cells and LX-2: 5 × 10^6^ cells/tumor) in a mixed Dulbecco’s modified Eagle medium (DMEM) (Nacalai Tesque Inc., Kyoto, Japan) and Matrigel solution (1:1) (Corning, Glendale, AZ, USA). The solution was administered subcutaneously to the backs of mice bilaterally, as previously described [27]. Two weeks thereafter, mice were divided into two groups (n = 6, each) and were injected with PBS or 0.5 mg/kg of LPS intraperitoneally twice a week, respectively. Two weeks later, the mice were sacrificed, and the tumors were harvested. The methods of this study were performed in accordance with the National Institutes of Health Guide for the Care and Use of Laboratory Animals (NIH publications number: 80–23), as revised in 2011. This study and all its experimental procedures were approved by the Animal Ethics Committee of Nara Medical University.

### 4.2. Cell Lines and Cell Culture

The human HSC line, LX-2, was purchased from Sigma–Aldrich Ltd. (Tokyo, Japan). Human differentiated HCC line, Huh7, HepG2, and JHH5 cells were distributed from the Japanese Collection of Research Bioresources Cell Bank (JCRB Cell Bank, Osaka, Japan). We also employed LPS from Escherichia coli. O55:B5 (Sigma–Aldrich, Tokyo, Japan). Recombinant Human EREG protein was purchased from R&D systems (Minneapolis, MN, USA).

Cell lines were cultured in DMEM, supplemented with 2% fetal bovine serum (FBS) (Biosera, Cholet, France) (used for LX-2) or 10% FBS (used for Huh7, HepG2, and JHH5), and 1% L-glutamine, penicillin, and streptomycin (Fuji Film, Tokyo, Japan) at 37 °C and 5% CO_2_. JHH5 cells were cultured in Williams’ E medium (Thermo Fisher Scientific, Waltham, MA, USA) with 10% fetal calf serum. For co-culture assays, Transwell™ Multiple Well Plates (24 mm inserts, TC-treated, 0.4 µm pore size, 6-well cluster plate) were used (Corning, Glendale, AZ, USA). Specifically, HCC lines were seeded in the lower compartment of the Transwell plates, whereas LX-2 was cultured in the upper inserts.

### 4.3. Cell Proliferation Assay

Cells were seeded in 96-well plates and, subsequently, treated with each reagent. After incubation, cell proliferation was assessed using the Premix WST-1 Cell Proliferation Assay System (Takara Bio, Kusatsu, Japan). The mean of the results was calculated from six replicates of each group, and the independent experiments were performed three times.

### 4.4. Cell Migration/Invasion Assays

Cell migration assays were performed using Transwell plates (8.0 µm pore size, 6-well plates) (Corning, Glendale, AZ, USA). Tumor cells were seeded into the upper insert at a density of 2.5 × 10^5^ cells/well in a serum-free medium. Serum-free medium containing 100 ng/mL of rEREG was added into the lower chamber as a chemoattractant. After 24 h of incubation at 37 °C, cells on the upper chamber were scraped to discord, and the cells invaded through the pores of the upper insert were stained with hematoxylin to count the mean number of cells in six random fields using a microscope. Similarly, a cell invasion assay using Transwell plates coated with Matrigel in the upper compartment was performed to count the number of invaded cells by stimulation with 100 ng/mL of rEREG added into the lower chamber.

### 4.5. RNA Isolation and Real-Time Quantitative PCR

Total RNA isolation was performed using RNeasy Mini kits (Qiagen, Hilden, Germany) for tumor tissues harvested from mice and cultured cell lines as per the manufacturer’s instructions. Cells were harvested independently from six wells in each group for the statistical analysis. Reverse transcription was performed using a high-capacity RNA-to-cDNA Kit (Thermo Fisher Scientific, Waltham, MA, USA). Real-time qPCR was performed using a SYBR™-Green PCR Master Mix (Thermo Fisher Scientific) and an Applied Biosystems StepOnePlus™ Real-Time PCR^®^ system (Thermo Fisher Scientific, Waltham, MA, USA). The primer pairs are listed in Table 1. The relative expression of each gene was normalized to *18S ribosomal RNA* expression and estimated using the 2^−∆∆^C_q_ method [49]. The expression levels are presented as the fold change relative to the internal control. 

### 4.6. Western Blotting

Whole-cell lysate was purified using a tissue protein extraction reagent supplemented with 0.1% proteinase and phosphatase inhibitors (Thermo Fisher Scientific, Waltham, MA, USA). Briefly, 15 μg of whole-cell lysates in each lane was separated with SDS-PAGE (10% NuPAGE Bis-tris gel) (Thermo Fisher Scientific, Waltham, MA, USA) and transferred to an Invitrolon PVDF membrane (Thermo Fisher Scientific, Waltham, MA, USA). PVDF membranes were subsequently blocked with 5% bovine serum albumin in Tris-buffered saline+0.1% Tween-20 for 1 h. Each membrane was subsequently incubated overnight at 4 °C with each antibody: EREG (1:2000, AF1195) (R&D Systems, Minneapolis, MN, USA), βactin (1:5000, ab8227) (Abcam, Cambridge, UK), EGFR (1:1000, 2232S), p-EGFR (Tyr1068) (1:1000, 2234S), T-ERK (1:1000, 9102S), and p-ERK (1:1000, 4370S) (Cell Signaling Technologies, Danvers, MA, USA), followed by incubation with horseradish peroxidase-conjugated secondary antibodies (1:5000) (GE Healthcare Life Sciences, Marlborough, MA, USA). Development was performed using Clarity Western ECL Substrate (Bio-Rad, Hercules, CA, USA) and detected by a CCD imager (Fusion Solo, M&S Instruments, Tokyo, Japan).

### 4.7. Detection of Angiogenesis Proteins

Cells were cultured with/without EREG, and the supernatant was subsequently collected after 24 h incubation. The measurement of secreted angiogenesis proteins in the culture supernatant was performed using the Human Angiogenesis Antibody Array-Membrane (20 Targets) (Abcam, Cambridge, UK) according to the manufacturer’s instructions. We prepared three membranes for the statistical analysis. Angiogenic factors included in this array-membrane is shown in Table 2. Development was performed using a Clarity Western ECL substrate and detected by a CCD imager. The intensity of the expression of each factor was measured using ImageJ software version 1.53a (National Institutes of Health, Bethesda, MD, USA).

### 4.8. ELISA Measurement

The EREG protein level in cultured supernatant was measured using a Human Epiregulin ELISA kit (DY1195-05, R&D Systems, Minneapolis, MN, USA) according to the manufacturer’s instructions. Standards and samples were pipetted into wells in a 96-well plate coated with anti-EREG antibody and, subsequently, incubated for 2 h. After incubation, the wells were washed, and a biotinylated antibody was put into the wells. To calculate the statistical differences, we prepared six samples for each group.

### 4.9. Immunohistochemistry/Immunofluorescence Staining

The resected tumor samples were fixed in 10% formalin overnight and, subsequently, embedded in paraffin. Five micro-meter sections of each block were deparaffinized and dehydrated on grass glides, followed by heat reactivation with citrate buffer (pH 6.0) for 15 min. The slides were blocked by PBS containing 5% BSA for 1 h. After the blocking procedure, the sections were incubated with each primary antibody overnight at 4 °C. The Vectastain Elite ABC kit was employed for detection after incubation with the primary antibodies as per the manufacturer’s indications (Vector Laboratories, Newark, CA, USA). Diaminobenzidine was used as a chromogen for visualization. For immunofluorescence staining, Alexa Fluor^TM^ 594 chicken anti-goat IgG (H + L) (Invitrogen, Waltham, MA, USA; A21468) and Alexa Fluor^TM^ 488 donkey anti-mouse IgG (H + L) (Invitrogen, Waltham, MA, USA; A21202) were used for the secondary antibody after overnight incubation with primary antibodies. The images of the tissue were captured using BZ-X710 (Keyence, Osaka, Japan).

### 4.10. Immunocytochemistry Staining

Cells were seeded on the chamber slides and cultured with each reagent. Cultured cells were fixed with 4% paraformaldehyde phosphate buffer solution (Wako, Tokyo, Japan) for 15 min at room temperature and, subsequently, permeabilized in PBS containing 0.5% Triton-X. Chamber slides were blocked with 5% goat serum in PBS with 0.1% Tween 20 (BMS, Tokyo, Japan), followed by incubation with an anti-EREG antibody (AF1195, R&D Systems, Minneapolis, MN, USA) overnight at 4 °C. Alexa Fluor^TM^ 488 goat anti-mouse IgG (H + L) (Invitrogen, Waltham, MA, USA; A11001) was used for the secondary antibody. The images of cells were captured using BZ-X710 (Keyence, Osaka, Japan).

### 4.11. Statistical Analysis

Statistical analysis was performed using Student’s *t*-test or a one-way analysis of variance using GraphPad Prism version 9.0 (GraphPad Software, La Jolla, CA, USA). Data are presented as mean ± SEM. Statistical significance was defined as follows: *; *p* < 0.05, **; *p* < 0.01, ***; *p* < 0.001, and ****; *p* < 0.0001, ns; not significant.

## 5. Conclusions

EREG derived from activated HSCs under the LPS stimulation is an important cytokine to exaggerate the development of HCC. In this study, EREG showed the promotion of the cell proliferation and migration/invasion of Huh7, an EGFR-expressing liver cancer cell line. It also induced a tumor angiogenic factor IL-8 by Huh7 with *EGFR* expression. In our mouse model, LPS resulted in accelerated tumor growth and increased neovascularization along with the increased EREG and IL-8 expression in xenograft tumors. Together with these data, EREG plays a crucial role in the progression of EGFR-positive HCC, meaning that targeting EREG therapy may contribute to developing a novel, tailor-made therapeutic strategy against HCC in the future.

## Figures and Tables

**Table 1:** Forward and reverse sequences of the primers used for RT-PCR.

Human	Forward	Reverse	Accession Number
*CDK1*	TTGGATTCTATCCCTCCTGG	CTGGAGTTGAGTAACGAGCTGA	NM_001786.5
*CDK2*	TGGTACCGAGCTCCTGAAAT	GAATCTCCAGGGAATAGGGC	NM_052827.4
*CYCLINA1*	CCGTGGAGTCTGAAGCAATG	CTCCTGTACTGCCCATTTGC	NM_001413923.1
*CYCLINB1*	GAACCTGAGCCAGAACCTGA	ACAGGTCTTCTTCTGCAGGG	NM_031966.4
*CYCLIND1*	CCGTCCATGCGGAAGATC	ATGGCCAGCGGGAAGAC	NM_053056.3
*CYCLINE1*	CGCTGATGAAGATGCACACA	ACAGAAGAGAACGTGGAGCA	NM_001322262.2
*CDK4*	CCCACACAAGCGAATCTCTG	ACCCTCCATAGCCTCAGAGA	NM_000075.4
*CDK6*	AGGCATTTTGGGAACTGTTG	TCCCATCCACTTCAAAGGAG	NM_001145306.2
*EREG*	ACTGGTGTCCGATGTGAACA	TTCAGACTTGCGGCAACTCT	NM_001432.3
*EGF*	CAGGGAAGATGACCACCACT	CAGTTCCCACCACTTCAGGT	NM_001963.6
*HBEGF*	TGGGAACTCACTTTCCCTTG	CAGCTCCAATGTTCCCTGTT	NM_001945.3
*18S rRNA*	AAACGGCTACCACATCCAAG	CCTCCAATGGATCCTCGTTA	NR_003286.4
*TGFA*	AATCCATCAGCAGGGATCTG	GATTTGGCCTGAAATGCCTA	NM_003236.4
*AREG*	TGGATTGGACCTCAATGACA	AGCCAGGTATTTGTGGTTCG	NM_001657.3
*BTC*	GCTCATTCATGCCCTTTCTC	AATTTCGAGAGCCACCATTG	NM_001316963.2
*IL-8*	TAGCAAAATTGAGGCCAAGG	AAACCAAGGCACAGTGGAAC	NM_000584.4
**Mouse**	**Forward**	**Reverse**	**Accession Number**
*18s rrna*	CGCGGTTCTATTTTGTTGGT	AGTCGGCATCGTTTATGGTC	NR_003278.3
*Cd34*	GGGTAGCTCTCTGCCTGATG	TCTCTGAGATGGCTGGTGTG	NM_133654.4
*F* *lt1*	TCACTCAGCGCATGGCAATA	CTCTCCTTCCGTCGGCATTT	NM_001159920.2
*K* *dr*	CAAGTGGCTAAGGGCATGGA	ATTTCAAAGGGAGGCGAGCA	NM_002253.4
*Pcam1*	GACGTGCAGTACACGGAAGT	GGAGCCTTCCGTTCTAGAGTAT	NM_000442.5

**Table 2:** Angiogenic factors listed in the array-membrane.

Angiogenic Factors Listed in the Array-Membrane							
Posi	Posi	Nega	Nega	Angiogenin	EGF	ENA-78	bFGF
Posi	Posi	Nega	Nega	Angiogenin	EGF	ENA-78	bFGF
GRO	IFN-γ	IGF-1	IL-6	IL-8	Leptin	MCP-1	PDGF-BB
GRO	IFN-γ	IGF-1	IL-6	IL-8	Leptin	MCP-1	PDGF-BB
PIGF	RANTES	TGF-β1	TIMP-1	TIMP-2	Thrombo- poietin	VEGF-A	VEGF-D
PIGF	RANTES	TGF-β1	TIMP-1	TIMP-2	Thrombo- poietin	VEGF-A	VEGF-D
Blank	Blank	Blank	Blank	Blank	Blank	Nega	Posi
Blank	Blank	Blank	Blank	Blank	Blank	Nega	Posi

## Data Availability

The original contributions presented in the study are included in the article, further inquiries can be directed to the corresponding author/s.
